# An overview of structural approaches to study therapeutic RNAs

**DOI:** 10.3389/fmolb.2022.1044126

**Published:** 2022-10-28

**Authors:** Luca Mollica, Francesca Anna Cupaioli, Grazisa Rossetti, Federica Chiappori

**Affiliations:** ^1^ Department of Medical Biotechnologies and Translational Medicine, L.I.T.A/University of Milan, Milan, Italy; ^2^ National Research Council—Institute for Biomedical Technologies, Milan, Italy; ^3^ IFOM ETS—The AIRC Institute of Molecular Oncology, Milan, Italy

**Keywords:** therapeutic RNAs, RNA structure, RNA binding, RNA dynamics and flexibility, RNA selectivity and specificity

## Abstract

RNAs provide considerable opportunities as therapeutic agent to expand the plethora of classical therapeutic targets, from extracellular and surface proteins to intracellular nucleic acids and its regulators, in a wide range of diseases. RNA versatility can be exploited to recognize cell types, perform cell therapy, and develop new vaccine classes. Therapeutic RNAs (aptamers, antisense nucleotides, siRNA, miRNA, mRNA and CRISPR-Cas9) can modulate or induce protein expression, inhibit molecular interactions, achieve genome editing as well as exon-skipping. A common RNA thread, which makes it very promising for therapeutic applications, is its structure, flexibility, and binding specificity. Moreover, RNA displays peculiar structural plasticity compared to proteins as well as to DNA. Here we summarize the recent advances and applications of therapeutic RNAs, and the experimental and computational methods to analyze their structure, by biophysical techniques (liquid-state NMR, scattering, reactivity, and computational simulations), with a focus on dynamic and flexibility aspects and to binding analysis. This will provide insights on the currently available RNA therapeutic applications and on the best techniques to evaluate its dynamics and reactivity.

## 1 Introduction

Modern molecular biology research redefined the central dogma that explains the flow of genetic information, from DNA to RNA and from RNA to protein (Crick, 1970) and shed the light on the multiple roles of non-coding RNA (ncRNA). The most familiar form of RNA is the protein coding RNA (mRNA), but only a small fraction of RNA molecules in cells (about 5%) belong to this class and the remaining are ncRNAs. They are strategic in cell biology: ncRNAs regulate gene expression and protein functions, catalyze chemical reactions, slice and dice genetic materials (including other RNAs), and take part in building proteins by transporting amino acids and linking them together (Mollocana-Lara et al., 2021). The large use of next generation sequencing, as well as bulk and single-cell gene-expression analysis, revealed new genome-based therapeutic targets (Paunovska et al., 2022). Besides, only 1.5% of the human genome encodes for proteins, and among them, less than the 15% displays a binding site targetable by small molecules (Hopkins and Groom, 2002; Ezkurdia et al., 2014). In this context, ncRNAs, have inspired scientists about how to harness RNA as medical treatment. The progress in RNA biology, bioinformatics, and nanotechnologies, mainly for the delivery vehicles, fostered the development of RNA-based therapies toward their translation in clinical practice. Expectations were met when US Food & Drug Administration (FDA) approved Patisiran, the first RNA interference (RNAi)-based treatment for hereditary transthyretin amyloidosis, and Givosiran, RNAi-drug for acute intermittent porphyria in 2018 and 2019 (Brown and Wobst, 2021). The potentiality of RNA-based therapeutic modalities has been recognized globally with the successful outcomes of SARS-CoV-2 virus (COVID-19) mRNA vaccine. To date, the majority of approved RNA-based therapeutics are antisense oligonucleotides (ASO), followed by siRNAs and mRNAs (Zhu et al., 2022). RNA-based therapies are currently suitable for pathologies with established genetic targets, such as cancers, immune diseases and Mendelian disorders and infectious diseases.

Therapeutic RNAs can modulate transcript level, inhibit RNA modulator activity, encode therapeutic protein, or induce alternative splicing, and also bind target proteins (Feng R. et al., 2021). Advantages of RNA-based therapies includes (I) acting on targets “undruggable” for a small molecules or proteins; (II) targeting a wide variety of cellular component including genes, transcripts, regulators and proteins at every level of cellular organization; (III) the high specificity, due to the base complementarity, unattainable with small molecules; (IV) the high purity of RNA construct; (V) rapid and lower cost effective development, by comparison to “traditional” small molecules or recombinant proteins based therapies; (VI) the possibility to personalize treatments, rapidly edit *ad hoc* the RNA construct sequence or to adapt to a new therapeutic request, such as pathogen variants.

Despite the advantages of RNA-based therapies, the development of RNA therapeutics meets challenges. RNAs are rapidly catabolized by ubiquitous RNases (Houseley and Tollervey, 2009) and exogenous RNAs induce acute immune response that cause cell toxicity. Furthermore, RNA therapeutics delivery represents another hurdle. RNA drugs cannot be orally administrated, their delivery system depends on the type of RNA-based therapeutics and targeting, and whether transient or stable expression is desired. RNAs are negatively charged and cannot cross cellular membranes or the blood brain barrier. The delivery of RNA therapeutics should guarantee RNA stability and give the possibility to reach target tissue, cell type or subcellular organelles at therapeutic concentration. The delivery method should help to solve these challenges, it must be biocompatible, reduce the immune activation and have higher delivery efficiency (Cupaioli et al., 2014). Development of nanoparticle addressed many of these needs as RNA therapeutics delivery system as outlined in (Paunovska et al., 2022).

RNA molecules fold, based on the Watson–Crick base pairing, into secondary structures and specific sub-structures, such as characteristic RNA loops, like hairpin loops, internal and external loops, base-pair stackings, multi-branch loops, bulge loops, junctions, pseudoknots, kissing hairpins, and so forth. Functions of ncRNAs as well as their effectiveness as therapeutic agents are deeply related to their folding because these structures are recognized by proteins, other RNAs and other parts of the same RNA. Their thermodynamic stability is crucial, and it is determined by conditions that occur and change when interacting with proteins or other ligands (Nowakowski and Tinoco, 1997). The prediction of stable optimal RNA secondary structures based on thermodynamic models, such as Turner’s nearest-neighbor model (Turner and Mathews, 2010), has some strategic significance to the development of RNA-based therapies, along with bioinformatics tools that help to predict RNA secondary structure by free energy minimization model as well as RNA modifications.

The implementation of therapeutics drug design and bioinformatics platforms, structural modeling and machine learning play a key role in this new RNA therapeutics field. Small molecule drug development pipelines that target enzymes, and protein–protein interactions cannot be applied to nucleic acids. The progresses in experimental resolution and computational modelling of RNA structures have enabled clinical translation of RNA-based therapy. In this review we will discuss the characteristic of RNA therapeutics and recent advances in this field, summarizing from the structural viewpoint, the available strategies, and the computational and experimental analysis methods.

## 2 Oligonucleotide therapies, recent advances

RNA-based therapies rely on both coding and non-coding RNAs, and mainly targets nucleic acids (ether DNA or RNA) and proteins. These therapies can control gene function by silencing or activation, splice modulation, transcript degradation, translational activation, or antigen synthesis, up to protein encoding and function, as also decrease or block protein production (Table 1). Aptamers are used to target proteins (Zhou and Rossi, 2016), while single-stranded antisense oligonucleotides (ASOs) and double-stranded molecules target nucleic acids by RNA interference (siRNA) (Watts and Corey, 2012). The mRNA can be administrated as therapy to promote transient protein expression and have recently evolved into mRNA vaccines and protein replacement therapies (Yu et al., 2020). RNAs are also used in genome editing for biological and therapeutics approaches, such as CRISPR-Cas gene editing (Zhang et al., 2021).

**TABLE 1:** Summary of therapeutic RNAs characteristics (ss, single strand; ds, double strand).

RNAs	Length	Target	Function	Strengths and weaknesses	
Aptamer (ss)	20–100 nt	Proteins, peptides, carbohydrates, and small molecules; extracellular, circulating, and intracellular	Agonist (activating target moleculesAntagonist, (blocking molecular interactions), Bispecific (recognize cell-type and target)	(+) small size, efficient design and synthesis, low production costs	(−) degradability
ASO (ss)	10–30 nt	mRNA, snRNA, miRNA, lncRNA, RNA binding proteins	Prevent or increase mRNA translation into protein, exon-skipping alters splicing process	(+) optimized design and synthesis protocol, high specificity, does not require auxiliary proteins	(−) transient effect
siRNA (ds)	20–25 nt (mature); 30–100 nt (precursor)	mRNA	Prevent mRNA translation into protein	(+) high specificity	(−) require auxiliary proteins
miRNA (ds)	22 nt	mRNA	Blocking translation or promoting degradation of the target by binding to the 3′ UTR; antimiRs (recognize endogenous miRNA) promote mRNA translation	(+/−) allowing multiple mismatches can have multiple targets, transient effect	
CRISPRsg RNA	10–12 nt sequence specificity	DNA	Gene editing	(+) restore damaged allele function	(−) delivery (must penetrate the nucleus), specificity is fundamental to prevent random targeting
mRNA (ss)	-	Multiple	Protein replacement therapy, vaccination, cell therapy	(+) relevant results as vaccine	(−) transient effect

### 2.1 Targeting proteins

#### 2.1.1 Aptamers

**FIGURE 1:**
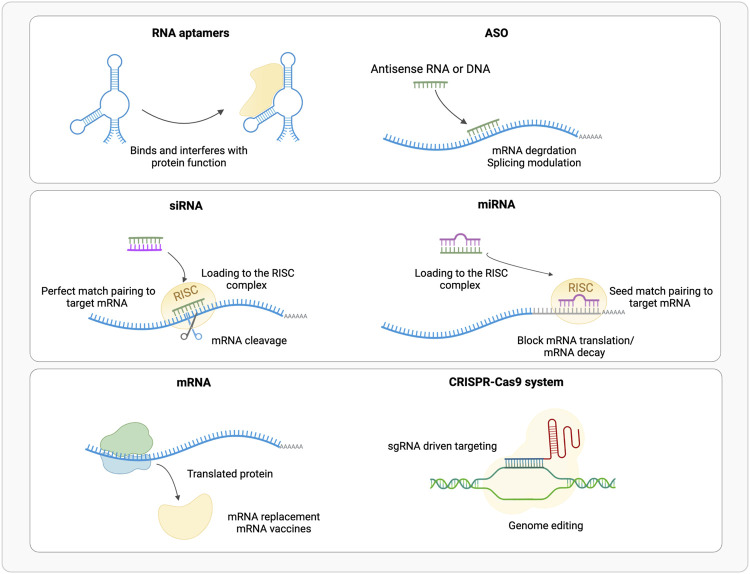
Schematic representation of the available RNA based therapeutic approaches (ASO, Antisense Oligonucleotide; RISC, RNA Inducing Silencing Complex). (Realized with Biorender).

Aptamers are short (20–100 nt) single strand nucleic acids that fold into specific tertiary structures (Figure 1). Aptamers binding specificity and affinity is the outcome of their tertiary structure rather than their sequence, like antibodies. Therefore, aptamers have potentially unlimited therapeutic targets, they can bind to a variety of extracellular, circulating, and intracellular targets: proteins, peptides beyond carbohydrates and small molecules (Mollocana-Lara et al., 2021) also allowing the recognition of specific cells and tissues. Aptamers can act as agonists, thus functionally activating their target molecules (Damase et al., 2021), and as antagonists thus blocking the interaction of molecules in pathways associated with disease development (Damase et al., 2021). Furthermore, aptamers can act as bispecific agents that recognize simultaneously two or more proteins, such as cell surface receptors, improving cell type targeting specificity (Zogg et al., 2022). Specificity can be improved by relatively small changes, as shown by the presence of a single methyl group that makes aptamer more sensitive by 10k-fold to caffeine than theophylline, which sterically prevent the formation of an H-bond with the RNA molecule (Jenison et al., 1994), and in cyclic adenine/guanine aptamer recognition where sensitivity goes as low as single base paring (Knappenberger et al., 2018).

In 1990 two different groups (Ellington and Szostak, 1990; Tuerk and Gold, 1990) developed the Systematic Evolution of Ligands by Exponential Enrichment (SELEX) technology to design and select aptamers with high affinity and specificity (Section 6.3).

Although aptamers display a similar function to antibodies, RNA aptamers have essential advantages: a smaller size, an efficient design method and chemical synthesis, performed entirely *in vitro*, with a very low variability among different batches, and lower production cost (Mollocana-Lara et al., 2021; Talap et al., 2021). The main issues are nucleases sensitivity, and the rapid excretion (Damase et al., 2021). As for other therapeutic RNAs, nucleic acid post-transcriptional and chemical modifications are useful to increase the structural stability, prevent RNase digestion and improve their functionality (Gehrig et al., 2012) Introducing afterwards chemical modification, though, can influence the aptamer tertiary structure, with consequent effects on target binding. Therapeutic aptamers are chemically modified to contain methyl groups at the 2′-position of their sugar moieties, rendering them resistant to degradation by serum nucleases and reducing the inherent immunogenicity of unmodified RNA (Odeh et al., 2019).

Structural studies and computational tools able to determine and predict aptamer structure and thermodynamic properties, before and after the target binding, are necessary to elucidate how small RNAs can fold into highly complex three-dimensional structures (Duchardt-Ferner et al., 2020). Moreover, the binding to the target highly influences aptamer final conformation, frequently inducing a transition toward a well-ordered structure (Hermann and Patel, 2000). Also, the identification of key interaction residues and structural motifs is helpful for aptamer design modification (Sharma et al., 2017). Actually, few *in silico* example are reported that determine the three-dimensional conformation of aptamers and the binding to their target (Oliveira et al., 2022).

### 2.2 Targeting nucleic acids

#### 2.2.1 Antisense oligonucleotides

ASOs are short (10–30 nt) single strand DNA or RNA molecules (Figure 1). Overall, ASOs activities can be ascribed to Watson-Crick base-pairing with their target sequences. ASOs prevent the mRNA translation into protein by pairing or steric hindrance (Baker et al., 1997), or alter its splicing (Hua et al., 2007), by blocking splicing cis-elements or affecting mRNA structure (Singh et al., 2018; Lim et al., 2020); few examples of enhanced mRNA translation upon ASO binding to upstream open reading frames (uORFs), are also available (Liang et al., 2016, 2017). Based on their mode of action, ASOs are classified into three categories: gapmers, steric blocking and exon-skipping. Gapmers are DNA stretches that bind the target RNA forming a hybrid double strand (DNA-RNA), that is recognized and hydrolyzed by endogenous RNase H (Wu et al., 2004; Lee et al., 2022). About 10 central nucleotides are necessary for the target recognition, flanked by shorter modified nucleotides. Sugar modification, not limited to gapmers, increases digestion resistance and binding affinity, and reduces immune activation, while phosphorothioate backbone promotes the transport into the nucleus (Eckstein, 2014; Shen et al., 2019) but triggers innate immune response (Fusco et al., 2019). Gapmers are efficient in downregulating nuclear targets, useful to treat diseases caused by an overexpression of toxic proteins, or mutated variants of the wild-type protein (Zogg et al., 2022). In addition, ASOs target single nucleotide polymorphisms (SNPs) associated with a mutant allele, maintaining wild-type allele function (Rinaldi and Wood, 2017).

Steric blocking ASOs are RNase H-independent and can inhibit or increase protein translation by binding to different targets. Inhibition is obtained by binding directly to mRNA and pre-mRNA or to trans-acting factors, like small nuclear RNAs, miRNA, long non-coding RNAs or RNA binding proteins (Quemener et al., 2020; Lee et al., 2022). This interaction prevents the binding to the AUG start codon, blocking the association of ribosomal subunits (Paunovska et al., 2022) or blocking polyadenylation sites, leading to shorter and destabilized transcripts (Vickers et al., 2001). Binding to the regulatory regions, uORF, instead, increases the translation of the main ORF by preventing the binding of translational suppressors (Zogg et al., 2022) up to 30–150% in a dose dependent manner (Rinaldi and Wood, 2017). MiRNAs that promote carcinogenesis and metastasis have also been targeted by ASOs (Shah et al., 2016).

Exon-skipping ASOs are single strand splice-switching oligonucleotides (SSOs) that recognize pre-mRNA altering the splicing process (Lee et al., 2022; Paunovska et al., 2022). SSOs are able to restore the correct function of aberrant splicing mutations causing disease, like Duchenne muscular dystrophy (Lee et al., 2022).

The nucleotide modifications mentioned above for gapmers, influence also ASOs solubility necessary for their delivery and cellular and nuclear membrane penetration, which remains the main challenge for these therapeutic molecules. Several methods can be employed to select an optimal candidate, including empirical testing of large numbers of mRNA complementary sequences, combinatorial arrays, computational RNA folding tools, and *in silico* prescreening using statistical modelling (Rinaldi and Wood, 2017; Fusco et al., 2019). The correct prediction of mRNA secondary structure allows to identify regions accessible to hybridization, necessary to design highly efficient ASOs (Chan et al., 2006). Recently targeted augmentation of nuclear gene output (TANGO) (Lee et al., 2022) has been proposed to prevent non-productive alternative splicing events, increasing the full-length functional transcript. TANGO strategy has been applied in the treatment of Dravet syndrome (Lee et al., 2022). Moreover, Li and colleagues employed three-dimensional structure in ASO design for targeting SARS-CoV-2 RNA frameshifting stimulation element (FSE), involved in the viral protein synthesis pathway, and transcriptional regulatory sequence (TRS) regions Li et al. (2021). The first one disrupts the FSE pseudoknot, an RNA secondary structural element, preventing the RNA-dependent RNA polymerase encoding. While the second one inhibits viral replication through TRS region; the ASO folds into a hairpin suggesting that the design of a highly specific ASO depends on its secondary structure.

#### 2.2.2 Small interfering RNAs

Small interfering RNAs (siRNAs) are short (20–25 nt) double stranded non-coding RNAs (Gavrilov and Saltzman, 2012), obtained from a precursor after a maturation process (Figure 1). The precursor of about 30–100 bp is processed by endogenous Dicer enzyme into 20–30 bp long siRNA; this includes two bases’ overhangs at the 3′ end single base paring in cyclic adenine/guanine aptamer recognition and interacting with RNA-induced silencing complex (RISC) to obtain mature interfering RNA (Damase et al., 2021). The RISC loaded antisense strand guides the complex to its target mRNA, where endonuclease argonaute 2 (AGO2) digest its phosphodiester backbone degrading the transcript (Feng R. et al., 2021; Mollocana-Lara et al., 2021). siRNAs recognized by AGO2 are fully complementary to their targets, while other AGO enzymes (AGO1, AGO3 and AGO4) catalyze endonuclease-mediated nonspecific mRNA degradation (Paunovska et al., 2022). Double stranded siRNAs are more effective than single stranded ASOs in downregulating mRNA targets (Zogg et al., 2022); RISC complex is localized in the cytoplasm rather than RNase H that is predominantly nuclear, thus target mRNAs at different stages of the transcriptional process (Lennox and Behlke, 2016). To obtain the desired siRNA, and not its antisense strand, the correct strand must be loaded into RISC; this can be achieved using asymmetric duplex, that will favor loading into RISC the strand with less stable hybridized end (Walton et al., 2010).

Like other therapeutic RNAs, nucleotide chemical modification in the sugar backbone prevents nuclease degradation, nucleoside modification to lower immunogenicity, increases safety, efficiency, and specificity (Layzer et al., 2004; Khvorova and Watts, 2017; Yu et al., 2020; Feng R. et al., 2021). The choice of the target site is critical to obtain an effective siRNA, which is usually localized closer to the start codon. Also, the C/G content influences the specificity and the stability of the siRNA, setting this content at least at 50%; while siRNA enriched in U-content have an immune-stimulatory effect (Yu et al., 2020). Moreover, a sequence motive AA_N_(19)__TT was suggested to be advantageous (Kurreck, 2006). A blast search to avoid possible unwanted pairing with other target genes is required. The thermodynamic stability of the siRNA duplex is a useful parameter to evaluate its functionality, concerning nucleotide position, principally for strand dissociation process, the beginning and the central portion of the siRNA should display a low stability, while the end should be thermodynamically more stable (Amarzguioui and Prydz, 2004; Reynolds et al., 2004; Ui-Tei et al., 2004). As for ASO, the target sequence on the mRNA must be identified in accessible regions to obtain efficient interference. Several bioinformatics tools are available to calculate thermodynamic and target secondary structure [Section 4.1 molecular dynamics, Section 6.5 docking paragraph and (Shabalina et al., 2006) for details] to help in siRNA design.

#### 2.2.3 microRNAs

Synthetic microRNAs (miRNAs) are short (22 nt) double strand non-coding RNAs (Figure 1), that regulate the expression of several mRNAs by blocking translation or promoting degradation of the target by binding to the 3′ untranslated region (UTR). Mature miRNA results from the processing of longer pre-miRNA molecules by Drosha and Dicer ribonucleases. Mature miRNA is loaded into RISC complex and miRISC recognizes the 3′UTR of target mRNA through 2 to7 bases match at miRNA 5′ seed region, thus inducing translational repression or mRNA degradation (Huntzinger and Izaurralde, 2011; Damase et al., 2021).

MiRNA-based therapeutic can mimic endogenous miRNAs or inhibit them (antimiRs): mimic miRNAs, administered as pre-miRNA, are double-stranded, match the endogenous miRNA and display the same activity restoring the lost miRNA expression in disease (Mollocana-Lara et al., 2021); miRNA inhibitors, administered as single-stranded oligonucleotides pair to endogenous miRNA blocking its activity (Zogg et al., 2022). AntimiRs with a 2′-O-methoxyethyl modification are also called antagomiRs (Rupaimoole and Slack, 2017).

MiRNAs are dysregulated into a wide variety of disease, therefore there is a clear interest for the use of miRNAs as therapeutic agents or targets (Yu et al., 2020; Chakraborty et al., 2021). Moreover, endogenous miRNAs target several genes simultaneously, frequently belonging to common pathway or cellular function. Indeed, miRNA-target complementarity rules allow for multiple mismatches targeting multiple mRNAs. The ability to act on multiple targets is, on the one hand, and an advantage, on the other hand, it reduces therapeutic miRNA specificity, making miRNA not suitable for personalized medicine. Moreover, miRNA effects are transient and require repeated administrations. However, comparing miRNA to other synthetic RNAs, therapeutic miRNAs display a reduced immune response (Zogg et al., 2022).

### 2.3 Encode for protein

#### 2.3.1 CRISPR single guide RNAs

**FIGURE 2:**
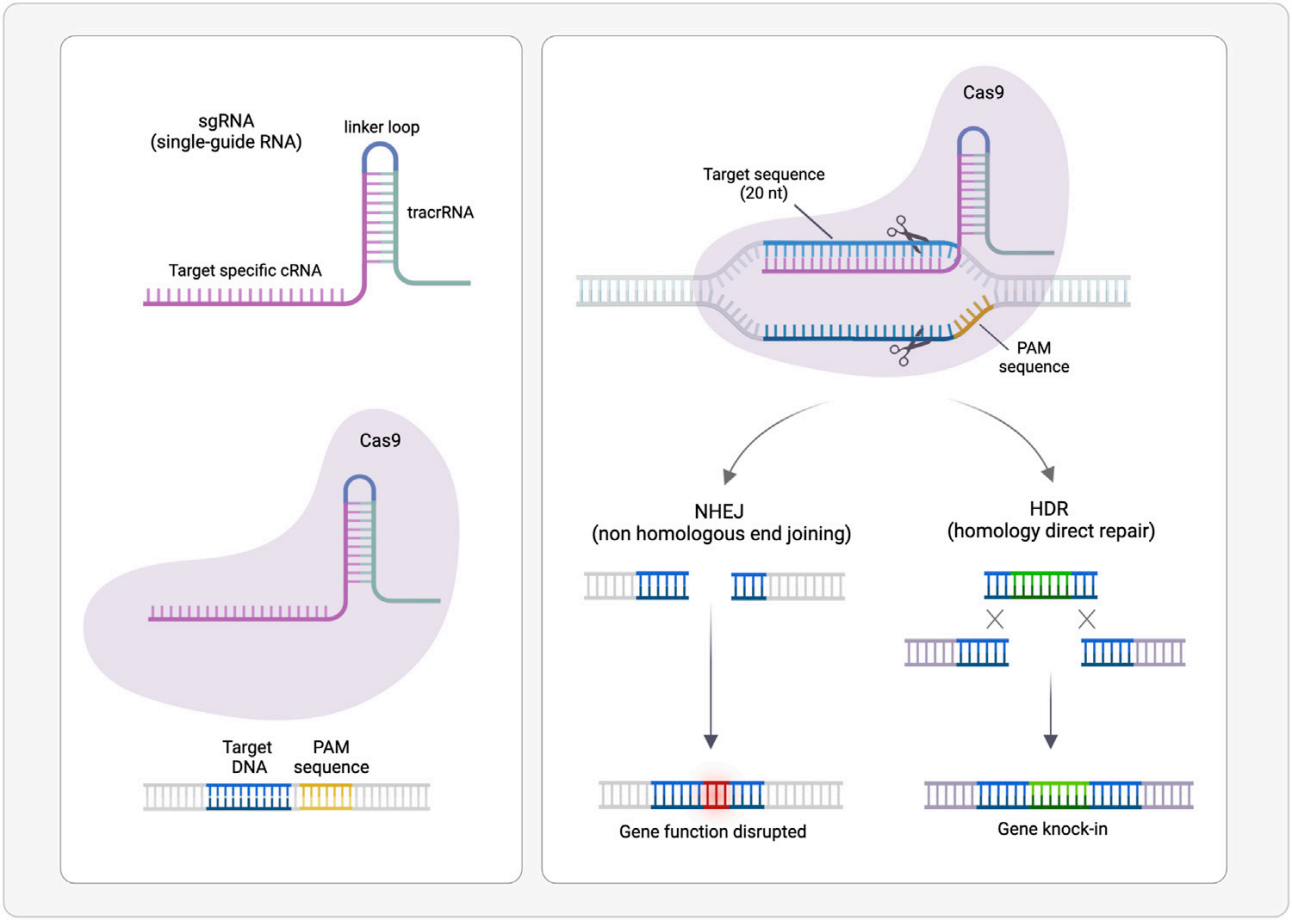
CRISPR/Cas9-mediated genome editing. A single guide RNA (sgRNA), consisting of a crRNA sequence that is specific to the DNA target, and a tracrRNA directs the Cas9 protein to a complementary sequence in the genome followed by a protospacer adjacent motif (PAM) where Cas9 will induce a double-stranded DNA cleavage. Repair after the DNA cut may occur *via* non-homologous end joining (NHEJ), leading to a random insertion/deletion of DNA, or homology directed repair (HDR) in the presence of a DNA repair template, which can be exploited to introduce precise genetic modifications or exogenous sequences. (Realized with Biorender).

CRISPR/Cas system belongs to the bacterial adaptive immune system that evolved to protect against phage infection by incorporating short repeats of the viral genome into the bacterial one. This system has been modified and used for genome editing accelerating scientific breakthroughs toward human gene therapy. CRISPR/Cas9 is used in mammalian cells and contains two components: a CRISPR-associated protein (Cas), a DNA endonuclease, and a single guide RNA (sgRNA) (Figure 1). In sgRNA the specificity-determining CRISPR RNA (crRNA) and an auxiliary trans-activating RNA (tracrRNA) are fused. The first ∼20 nucleotides of the crRNA include the seed sequence and a non-seed sequence. The seed sequence determines Cas specificity and is composed by the 10–12 bases complementary to the target DNA sequence, followed by a sequence called protospacer adjacent motif (PAM), a short (2–6 bp) in the genomic DNA. The PAM is essential for cleavage by Cas nuclease. The crRNA binds to the Cas9 through its hairpin scaffold and the sgRNA recognize with high specificity the PAM complementary sequence. Perfect complementarity between seed sequence included in crRNA and the target DNA is required. This direct Cas nuclease to the target sequence, where tracrRNA portion of sgRNA forms an R-loop due to the base paring with Cas-DNA. Upon RNA-DNA heteroduplex formation the nuclease introduces site-specific breaks into the 20-nucleotide DNA target sequence. The damaged DNA is repaired *via* non-homologous end joining (NHEJ) or homology-directed repair (HDR), thereby resulting in gene disruptions and inactivation of the targeted gene (Zhang et al., 2015; Jiang and Doudna, 2017). NHEJ is exploited also for linear DNA fragments tag-guided insertion (Pickar-Oliver and Gersbach, 2019) (Figure 2).

Cas nuclease specificity relies on the distance from PAM sequence to the cleavage site and on the capacity to perform double- or single-stranded breaks. For example, the most employed Cas9 performs double breaks three bases upstream PAM. CRISPR-Cas is at the cutting edge of genetic engineering: CRISPR-Cas9, has been modified to correct disease-causing mutations, inactivate oncogenes or activate oncosuppressors and to act as transcriptional or epigenetic regulator (Larson et al., 2013; Qi et al., 2013).

Clinical trials based on CRISPR gene editing are ongoing to treat genetic disorders such as cystic fibrosis, Duchenne muscular dystrophy, viral infections, immunological disorders, and cardiovascular diseases (Feng R. et al., 2021). The design of sgRNA is crucial to minimize off target cutting and therefore off-target mutations and cleavage; several bioinformatic tools are available for the rationale design of highly active and specific sgRNAs (Leonova and Gainetdinov, 2020).

The main challenge of this technology is the delivery to target cells because both the Cas protein and the single-guide RNA (sgRNA) must be present at sufficient concentrations to form intracellular ribonucleoproteins (Paunovska et al., 2022); and differently from the previously described RNA therapies, a DNA-target “drug” must penetrate both, cytoplasmic and nuclear membranes (Damase et al., 2021). As for previously presented RNA therapies, nucleotide chemical modifications can be useful to protect against RNase digestion and avoid immunogenicity; moreover, chemically modified gRNAs have been shown to enhance genome editing efficiency and target specificity in mammalian cells (Yu et al., 2020).

The fine-knowledge of this large complex, obtained by biochemical and structural studies on Cas9 at different stages of DNA target surveillance, provides key hints for its customization and application in therapies (Jiang and Doudna, 2017). Recently molecular dynamics (see paragraph Section 5.1 for method details) (Palermo et al., 2017), also coupled with FRET (Palermo et al., 2018) or solution NMR experiments (see paragraph Section 4.1 for method details) (Nierzwicki et al., 2021), have been applied to evaluate the conformational dynamics of the complex, necessary for catalytic activity, as well as the binding features of the single-guide RNA and of DNA-target (Mitchell et al., 2020). Studies at atomic details contribute to rational design of Cas9 proteins and to understand the sensibility of this “machine”, allowing a more effective design of guide-RNAs, thus obtaining a more efficient CRISP-Cas9 genome-editing tool.

#### 2.3.2 mRNA

The use of exogenous mRNA to mimic cellular mRNA to produce desired proteins is an established technology since many years (Damase et al., 2021) (Figure 1). Therapeutic mRNAs are much bigger in size than other therapeutic small RNAs as they maintain the same structure than the natural one: a single strand molecule, with a central coding sequence (CDs), flanked by a 5′ cap for nuclear export and translation promotion, a 5′UTR for ribosome recruitment, a 3′UTR for post-transcriptional control and a 3′ poli (A) tail for mRNA stability. mRNA can be employed for several therapeutic purposes: 1) protein replacement therapy, where mRNA is administered to compensate for a defective protein, or to supply therapeutic proteins; 2) vaccination, where mRNA encoding specific antigen(s) is administered to elicit protective immunity 3) cell therapy, where mRNA is transfected into the cells *ex vivo* to guide differentiation or alter cell phenotype, and then these cells are injected back to the patient (Chabanovska et al., 2021; Damase et al., 2021).

Therapeutic mRNAs have a short half-life; once reached the cytoplasm of target cells, they are digested in few hours (Damase et al., 2021). SARS-CoV-2 mRNA-based vaccines have promoted a further and fast in the development of the technology (Corbett et al., 2020; Patel et al., 2022). These mRNA vaccines encode the prefusion stabilized full-length spike protein (Baden et al., 2021) with relevant results in disease control.

As previously described therapeutic RNAs, also mRNA solubility, efficacy, and immunotolerance, can be improved by chemically nucleotide modification, as well as by “converting” mRNA into circular RNA (circRNAs) (Mollocana-Lara et al., 2021).

## 3 A chemical introduction to RNA structure and dynamics

A strategic role in the development of RNA-based therapies is played by physical-chemistry, structural biology, and computational modelling of biomacromolecules. These disciplines ensure the fine tuning and/or prediction of the intramolecular interactions that drive molecular recognition. Hence, mastering the RNA chemistry is critical in this respect. Despite the apparent chemical similarity between DNA and RNA nucleotide monomers sidechains, the subtle difference between the two backbone chemical moieties enormously influences both the reactivity and the dynamics of the two polymers. Each nucleotide consists of a planar aromatic base attached to a furanose ring with a 5′-phosphate group. Phosphodiester linkages between successive sugar residues groups (linking the 3′-carbon of each sugar to the 5′-carbon of the next one) drives the polymerization of RNA chains, leaving a free 5′-position at one end of the chain and a free 3′-position at the other. If this process is basically the same for DNA, the 2′-OH group of the RNA ribose (2′-H in DNA) moiety makes RNA less chemically stable than DNA by facilitating self-cleavage reactions, thus making RNA more flexible and prone to adaptation in cells. Moreover, 2′-OH group is a versatile hydrogen bond donor and acceptor, allowing the formation of unique macromolecular spatial arrangements with a functional role. This is reflected by the necessity of describing RNA molecules hierarchically by analyzing both their secondary structure, i.e., the interaction between nucleic acids regardless their three-dimensional orientation and the overall folding, and their tertiary structures, i.e., the spatial organization of atoms belonging to the RNA polymer. Moreover, post-translational modification of RNA has an impact on topological and spatial arrangement of RNA molecules (Nachtergaele and He, 2017).

Secondary (2D) structure is defined by the pattern of Watson−Crick canonical base pairs (A-U, C-G base pairs). Canonical double helices alternate with unpaired regions, namely that do not form canonical base pairs. RNA topology is determined by the backbone, which depends on the position and direction of the backbone segments attached to the bases. This links a local property (base pair geometry) with the global topology, which determines the molecule’s biological function. Building on the basic architecture defined by the 2D structure, RNA 3D structure is determined by some canonical and many noncanonical base pair interactions that often involve H-bonding through the 2′-OH group. RNA folding kinetics is hierarchical and sequential secondary (2D) structure motifs display an overall higher stability compared to 3D structures, they fold autonomously, and usually (Brion and Westhof, 2003) before and independently of 3D structure. The description of the RNA formal structures is completed and complicated by pseudoknots, i.e., an extended canonical base pairing between a hairpin loop a 2D element and a single distal complementary strand region.

A single RNA molecule, with a well-defined sequence, often has multiple accessible 2D and 3D structures that lie within a narrow range of folding free energies. These are sampled because of thermal fluctuations or proteins and other cofactors interactions induce or select specific RNA conformations. The time scales over which these alternate structures form and disappear can range from microseconds for simple base pairing changes to seconds (or even longer) for complex 2D refolding events (Al-Hashimi and Walter, 2008). Because RNA molecules often play multiple roles in biological processes, their native states can be anything from structured folded architectures to intrinsically disordered dynamical single-stranded ensembles.

## 4 Experimental methods for the study of RNA dynamics and flexibility

**FIGURE 3:**
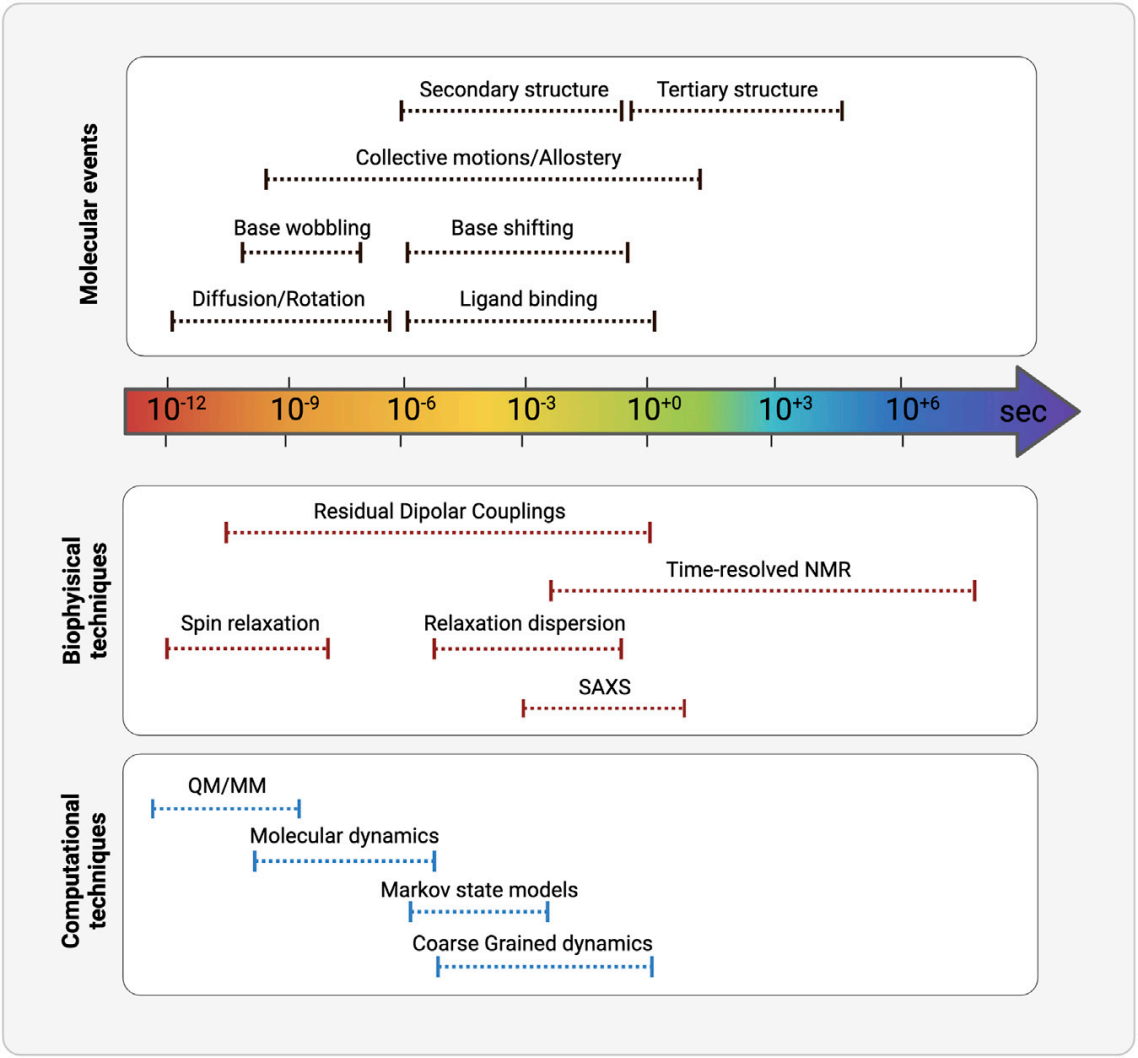
Schematic representation of the timescales overlaps between (from the top) molecular events, biophysical/analytical techniques and computational methodologies. (Realized with Biorender).

After at least three decades of investigations led by physico-chemical techniques, the structure-function paradigm is currently integrated in almost all the studies that address how biomacromolecules mechanistically exert their function. More recently the role of the internal motions has been elucidated and has revealed to be crucial in molecular recognition between partners. Macromolecular structures obtained by X-ray crystallography, high-resolution NMR spectroscopy and from cryoelectron microscopy constitute an essential technique for characterizing the lowest (i.e., the most probable conformation) energy state of a molecular system and understanding molecular recognition. Nowadays, structural plasticity has become a stable asset to understand macromolecular functionality. The combination of complementary physico-chemical techniques (either experimental or a combination of experimental and computational techniques) allows to extensive investigate structural equilibrium of molecules, also to obtain truthful models. It is of key importance also the experimental characterization of the less populated states [e.g., base-pairing partner switches for RNA (Dethoff et al., 2012)], that are in thermal equilibrium with the ground state of any macromolecular system and can be now assessed by the new methodologies (Figure 3; Table 2).

**Table 2:** Summary of biophysical techniques to study RNA complexes.

Technique		Information content	Strengths	Weaknesses
Nuclear Magnetic Resonance	Real time NMR	Time resolved description of chemical processes	Easily implemented	Overcrowded spectra from moderately large to big macromolecules
	Relaxation	Time resolved description of molecular motions, detection of transient states	Well established and documented method, spectroscopic determination of interconversion kinetics between populations	Labelled samples, complex computational treatment
	Residual Dipolar Couplings	Time-averaged orientation of macromolecular regions/domains	Average of a wide family of timescales	Labelled samples, complex computational treatment
Scattering	SAXS	Molecular shape, domains rearrangement	Liquid sample (no crystals)	Access through facilities, low distinguishability of proteins and RNA
	SANS	Molecular shape, domains rearrangement	Liquid sample (no crystals), distinguishability of proteins and RNA through contrast experiments	Access through facilities
Reactivity	SHAPE	Detection of nucleotides in flexible regions	Easy sample treatment, application in living cells, connection to structures and dynamics	Complex computational treatment, inference
	DMS	Detection of non-paired nucleotides and/or fall in flexible regions	Easy sample treatment	Purine specific
Simulations	Molecular Dynamics (MD)	Pico-micro-second dynamics of macromolecules	Well established and documented method	Minimum trapping, limited timescale
	Replica Exchange MD	Deep exploration of the free energy surface	Adaptable schema, coupling of precision and accuracy, unsupervised simulations	Computationally demanding
	Metadynamics	Deep exploration of the free energy surface	Coupling of precision and accuracy	Computationally demanding, choice collective variables for complex systems
	Coarse Grained	Nano-milli-second dynamics of macromolecules	Explored timescales greater than other methods	Low precision

In this section we will discuss biophysical methods that are now routinely used for the characterization of the interaction between biomolecules and their partners, either other macromolecules or small molecules, with a focus on their application to RNA. In this respect, due also to the relatively recent role of RNA molecules as drugs, these techniques will be reviewed regardless the oligonucleotides function as ligand or as target, with the aim of highlighting the general principles behind the characterization of their interaction with any partner. Moreover, in this respect a purpose of this review is the suggestion of the application of these techniques, once they have not been intended in this way, to the world of RNA-based drug discovery.

### 4.1 Experimental dynamics: Nuclear magnetic resonance in the liquid state

Nuclear magnetic resonance (NMR) spectroscopy is the most advanced experimental technique that can efficiently provide details ranging from atomic positions to inner or global dynamics (Hodgkinson and Emsley, 2000; Lotsch et al., 2007). The inner dynamics and global hydrodynamics of any molecule influence the NMR observables, allowing the determination of phase changes, conformational and configurational alterations, solubility, and diffusion potential (Levitt, 2008). If this is an asset of protein-bases systems, from the 80s, the widespread application of NMR spectroscopy for the characterization of nucleic acids under solution condition is more recent. Higher magnetic fields and cryogenic technologies partially overcome the limitations deriving from low chemical heterogeneity of RNA building blocks (only four nucleotides, compared to 20 amino acids), leading to low chemical shifts dispersion of nuclei of interest (i.e., closeness of resonances, hence low distinguishability).

#### 4.1.1 Real time nuclear magnetic resonance

This technique monitors RNA dynamics in real-time (i.e., a series of spectra recorded at regular intervals), and is the most intuitive NMR-based method, as the changes that occur in a biological system can be directly associated with disappearance and appearance of signals. In a classical drug discovery context (i.e., small-molecules based) kinetic properties of non-equilibrium ligand-dependent RNA can be followed using time-resolved NMR (Buck et al., 2009; Lee et al., 2010). Real time NMR is used to investigate RNA conformational transitions, such as RNA (re-)folding, the catalytic reactions of ribozymes or purine riboswitches started by addition of the ligand (Buck et al., 2009; Steinert et al., 2017) or in some cases also RNA binding affinity for cations (Müller et al., 2021). Recently, successive NMR measuring is used to investigate the dynamic adaptation of RNA modifications in response to environmental changes. In Barraud et al. (2019), the authors describe the maturation of yeast tRNAPhe in cell culture extracts using labelled isotype in a continuous and time-resolved fashion. A general and extensive overview of real-time NMR spectroscopy methods, with an interesting focus on folding-inducing ligands, is reported in Pintér et al. (2021).

#### 4.1.2 Residual dipolar couplings measurements and relaxation dispersion

The proper understanding of biomolecular recognition mechanisms that take place in drug targeting is of paramount importance to improve the efficiency of drug discovery and development. The flexible nature of RNA influences the molecular recognition mechanisms and its characterization plays a key role in a drug discovery context, i.e., allowing the identification of multiple conformers involved in the process. NMR spectroscopy offers a wide range of techniques that can be informative in this context also including time resolution of structural interconversion.

Most of current NMR techniques for the investigation of biomolecules structure and dynamics is based on isotope labelling, i.e., the enrichment of NMR-sensitive isotopes (^13^C, ^15^N) (Barton et al., 2013). As extensively reviewed in Lu et al. (2010), the three main methods for isotope labelling of nucleic acids are chemical synthesis, enzymatic synthesis using T7 RNA polymerase and segmental labelling. Although modified NTPs can readily be incorporated into specific positions, low coupling efficiencies limit chemical synthesis to relatively small RNAs. RNAs for NMR-based structure determination are now commonly prepared by *in vitro* transcription using the T7 RNA polymerase (RNAP) enzyme (Belin, 1997). Recently, segmental labeling approaches have been developed for studying sub-domain structure in the context of intact, functional RNAs (Lu et al., 2010), leading to an efficient method for overcoming signal overlap problems associated with larger RNAs. Labelling strategies are the crucial step for assigning the resonances of atomic species within a macromolecule according to their chemical nature (chemical shifts) (Lu et al., 2010), as well for performing dynamics-related measurements such as spin relaxation and residual dipolar couplings (RDCs).

Spin relaxation, that can be seen as a measurement of the decay of the excited magnetization perpendicular to an externally applied magnetic field, has been widely used in the last two decades for the characterization of internal protein motions. The theoretical framework for its interpretation correlates the macromolecule overall shape, reference structure and hydrodynamics to its relaxation properties. While this provides a proper description of the relaxation-related observables for globular proteins, its application to flexible objects like intrinsically disordered proteins (Salvi et al., 2017) or RNA has been more difficult. For this reason, protein-RNA binding has been often studied by means of spin relaxation experiments that could detect the conformational recognition of RNA on the surface of proteins (Wurm et al., 2016; Lixa et al., 2018). Relaxation dispersion is suitable for investigating transition/transient states in structures, often reported as invisible states. Relaxation dispersion can be observed in systems that interconvert between two distinct states at a rate that is comparable to the difference between the frequencies detected for the two states: the exchange causes significant line-broadening of signals from both states. This technique is suitable for detecting the RNA molecules dynamic states as well as the effect of small ligands on them, allowing the distinction between induced fit and conformational selection mechanism (Vogt and Cera, 2012). In Orlovsky et al. (2020) authors highlighted conformational penalties as major determinants of RNA-ligand binding affinity as well as a source of binding cooperativity, with important implications for a predictive understanding of how RNA is recognized and for RNA-targeted drug discovery. The idea of preformed binding site for a ligand is also suggested by another recent study (Ding et al., 2019) demonstrating (using relaxation data and free electron laser X-ray diffraction experiments) that the local timescales and entity of motions in the binding site for riboswitch aptamer is comparable in the free and complexed form of RNA. The same approach has been adopted to RNA considered as a ligand and applied to Fsu preQ1 class I aptamer for the determination of the binding kinetics of the ligand preQ1 (Moschen et al., 2015). Interestingly, the study revealed aside the kinetic parameters of binding that the RNA molecule adopted a pseudoknot structure prior to binding and in slow conformational exchange with the unknotted form, revealing how in this class of molecules the approaches used for revealing the overall shape and structure is mandatory for a complete characterization of binding.

Chemical bonds and structural motifs orientation have an influence on dipolar couplings (DCs), i.e., the spectroscopic observable that reflects the interaction between individual nuclear spins energy levels as mediated by their distance, electronic environment, and relative orientation. Residual dipolar couplings (RDCs) are variations of complete motional averaging of DCs due to a partial molecular alignment to the external magnetic field. RDCs can also provide information about dynamics on micro-milliseconds timescale. An application of RDCs for the determination of RNA binding modes to proteins has been reported in Borkar et al. (2016) once the structure of the complex formed by the HIV-1 protein transactivator of transcription (Tat) and its cognate transactivation response element (TAR) RNA transactivates viral transcription, a paradigm for the widespread occurrence of conformational rearrangements in protein-RNA recognition. The free energy landscape (FEL) of the complex has been determined using NMR residual dipolar couplings in replica-averaged metadynamics simulations (see further), showing the coexistence of three low energy states, comprising the absolute minimum and two intermediates (the one with the highest energy corresponding to the transition state of the entire system). A campaign of high-throughput screening against ∼100,000 drug-like small molecules (Ganser et al., 2018) has been performed on this target, revealing again the superiority of RDC-mediated ensembles that enrich libraries with true hits. Chang et al. (2020) resolved with the help of RDCs the structure of a microRNA (miRNA) that contains the tandem UU:GA base pair mismatch and used it for a drug-discovery purpose. This mismatch motif occurs in the helices of large rRNA subunits of several organisms, including human pathogens like HIV (Bilbille et al., 2009), making it an optimal target for pharmaceutical purposes. Due to the presence of the mismatch, the structure is very flexible, and it has been resolved with an extensive usage of molecular dynamics (MD) (see further) simulations and further refined with residual dipolar coupling constants to generate an ensemble of structures against which a virtual screening of 64,480 small molecules was performed to identify candidate compounds specifically bound by the motif. This allowed the identification of a single compound (2-amino-1,3-benzothiazole-6-carboxamide, ZN423), which binds the miRNA with moderate affinity but includes the flanking A-U and G-C base pairs in the interaction. These base pairs contribute to the structural and/or dynamic features necessary to ZN423 binding. Moreover, the finding that specificity for the UU:GA mismatch is dependent on the flanking sequence demonstrates the importance of context effect and increases the possible number of small non-canonical features that can be specifically targeted.

#### 4.1.3 Small angle X-rays scattering and neutrons scattering.

Small Angle X-rays scattering (SAXS) provides an accurate and diverse set of parameters that describe biomolecules, including global information about macromolecular size and shape, intermolecular association, domain motion and linker flexibility (Table 2). Recently, SAXS emerged as a prominent technology to facilitate research and quality control at different steps of drug development, i.e., providing a fingerprint of complexes formation, as well as of drug delivery systems. Being sensitive to the complex size, SAXS is particularly suited for the characterization of large drug molecules, like RNA, allowing the analysis of single components signals as weighted shape/structure.

During SAXS experiments, X-rays are scattered at the electrons of the inner atomic shells. Upon interaction a spherical wave is emitted with conserved energy and wavelength and its intensity is proportional to the electronic density at nuclear position within a structure. Such intensity can be interpreted *via* readily available computational tools to model structures from SAXS data (Chen and Pollack, 2016); more recently developed computational methods can be applied to model the complex solution environment around RNA (Nguyen et al., 2014; Yang, 2014). Similar principles are at the basis of small angle nuclear scattering (SANS), in this technique neutrons are scattered by nuclei and substantially exhibit a wave-like behavior (Lapinaite et al., 2020). Isotopes of the same element can have very different neutron scattering properties, allowing contrast experiments, i.e., the replacement of H_2_O with D_2_O (deuterated water). Contrast variation methods for neutron scattering are perhaps more effective to implement as they involve replacing H_2_O with D_2_O and enable “blanking” of either the protein or nucleic acid components of the sample. Contrast variation SAXS presents unique opportunities for studying the dynamics of RNA–protein complexes, for example adding sucrose to the buffer to increase its electron density to equal that of the protein, ensuring significant scattering of the RNA even above this electron dense background. The contrast variation method has recently been applied to study DNA–protein complexes, where the use of high intensity X-ray sources enables time resolved studies (Chen et al., 2014).

SAXS can be used to investigate dynamics of nucleic acids, except for the experimental characterization of complex formation and hydrodynamics. Dynamics of biological relevant nucleic acids has been investigated (Chen et al., 2014) building models of nucleosome core particles (NCPs) based upon time-resolved SAXS experiments, that addressed the symmetry of DNA release from the histone core and the interaction of histones with the DNA during unwrapping. This study clearly revealed the asymmetric nature of NCPs disruption and provided a bead models of DNA spatial organization, and an estimate of a timescale (200 ms) for the residence time of proteins in contact with DNA molecules. The vast majority of SAXS-based studies performed on nucleic acids are coupled with complementary experiments that provide dynamic details of the complexes formation, mainly MD (see further), mass spectrometry and nuclear magnetic resonance spectroscopy (Szameit et al., 2016; MacOšek et al., 2021). The usefulness of SAXS as an analytical tool for the characterization of RNA as drug has been demonstrated by Szameit and colleagues, who reported a study about protein-interacting RNA aptamer (Szameit et al., 2016). They studied the structure and target interaction properties of two RNA aptamers, AIR-3 and its truncated form AIR-3A, specific for human interleukin-6 receptor (hIL-6R), a key player in inflammatory diseases and cancer, recently exploited for *in vitro* drug delivery studies. AIR-3 (with its variants incorporating gemcitabine) and AIR-3A were investigated by different methods including RNA structure probing, SAXS and microscale thermophoresis. SAXS experiments allowed the determination of the overall shape (hence, partially, of dynamics) of self-recognition during AIR-3/A assembly’s formation, providing an indication of its dimeric state in the apo- and -holo- forms with the help of MD simulations.

#### 4.1.4 Single nucleotide reactivity and dynamics/accessibility: SHAPE and DMS

Chemical reactivity of flexible molecules is influenced by inner dynamics, and it is also altered by binding. In the last decade, RNA profiling is employed for characterizing the inner dynamics and topology of RNA, both as single molecule and during molecular recognition. This is challenging, but a promising development in the field of RNA-based drug discovery, offers the possibility to comparatively monitor the effect of RNA binders, flexibility, and selectivity, including several quantitative interpretations of reactivity, in a relatively fast and direct way.

RNA chemical probing is a versatile technique that can be used to elucidate RNA secondary and tertiary structures as well as RNA-ligand interactions at single-nucleotide resolution in the solution state (Stern et al., 1988; Waduge et al., 2019) (Table 2). Specific positions in a folded RNA are exposed to chemical agents, according to the local conformation and/or base-pairing patterns. Chemical probing can be broadly categorized into two groups depending on the nature of the chemical reaction of the probing agent: 1) base-specific attacks that are predominant in single-stranded regions of the folded RNA, and 2) non-base-specific attacks that are due to the ability of nucleotides, particularly in flexible regions of the folded RNA, to sample favorable conformations. Among the known chemical probes, dimethyl sulfate (DMS) is a particularly useful base-specific reagent that covalently modifies both purines and pyrimidines methylating N1 position of adenine, N3 position of cytosine and N7 position of guanine. Therefore, DMS can be used to examine Watson-Crick base-pairing accessibility of adenine or cytosine, or Hoogsteen base-pairing accessibility of guanine in solution. In addition, DMS can be utilized in a broad pH range and other solution conditions without effects on the reactivity. Regions with specific secondary structures within a folded RNA molecule can be identified by studying the DMS modification patterns, also as a function of different environment conditions (Peattie, 1979). Otherwise, DMS probing is very useful to identify regions within folded RNA that change conformation or are protected upon ligand binding, allowing the mapping of protein-RNA or RNA-RNA interactions DMS-based chemical probing supports the determination of secondary and tertiary structures of nucleic acids, while the information about the internal dynamics and accessibility of single nucleotides is normally conveyed by SHAPE (selective 2-hydroxyl acylation analyzed by primer extension) chemistry (Smola et al., 2015). Some studies (Gherghe et al., 2008; Hurst et al., 2018; Mlýnský and Bussi, 2018) suggested that SHAPE reactivity is correlated with nucleotide flexibility and to the extent to which a nucleotide is constrained by base pairing or other interactions. The RNA secondary structure prediction problem is reformulated using quantitative nucleotide-resolution SHAPE information in concert with thermodynamic parameters for RNA folding, by placing effective constraints on the possible structures in a conformational pool generated by computational modeling software (Zhou et al., 2021). Interestingly, SHAPE and DMS reactivities have demonstrated to be similar regardless of the probing method, especially for long non-coding RNAs [lncRNA, e.g., Malat1 and Braveheart (Bvht)].

SHAPE-MaP is a further extension of the SHAPE reactivity coupled with the mutational profiling of the RNA under investigation. The approach exploits conditions that cause reverse transcriptase to misread SHAPE-modified nucleotides and incorporate a nucleotide noncomplementary to the original sequence in the newly synthesized cDNA. The positions and relative frequencies of SHAPE adducts are thus immediately, directly, and permanently recorded as mutations in the cDNA primary sequence, thereby creating a SHAPE-MaP. In a SHAPE-MaP experiment the RNA is modified under denaturing conditions to control for sequence-specific biases in detection of adduct-induced mutations. This technique has revealed its power and flexibility in different directions ranging from the structural mapping of RNA of known structure to the topological analysis of an integral genome. The former analysis has been performed on *Escherichia coli* thiamine pyrophosphate (TPP) riboswitch aptamer domain in the presence and absence of saturating concentrations of the TPP ligand: SHAPE-MaP confirmed the known reactivity pattern for the folded, ligand-bound RNA and accurately reported nucleotide-resolution reactivity differences that occur upon ligand binding. The latter has been performed on the HIV-1 RNA genome, thus updating, and completing its high-resolution topological model. An extension of this technology has been proposed for its performance in live cells (Smola and Weeks, 2018): it can be applied in cell types ranging from bacteria to cultured mammalian cells and is compatible with a variety of structure-probing reagents, informing new biological hypotheses and emphasizing downstream analyses that reveal sequence or structure motifs important for RNA interactions in cells.

A recent publication (Kim et al., 2020) proposed the use of SHAPE and DMS reactivity and of SAXS for determining the topology, the structure and the binding property of the 660 nucleotides long non-coding RNA (lncRNA) Bvht involved in the epigenetic control of cardiac development. This approach partially answers to the difficulty to predict large RNA structures due to the coexistence of several similar-but-different structures within 1–2 kcal/mol and to a strong influence of kinetic processes and/or protein–RNA interactions that may modulate RNA folding. Noteworthy, SHAPE/DMS results are used as spatial restraints to model the dynamic behavior of RNA at atomic level; besides a model of interaction between the regulator protein CNBP and Bvht, with the AGIL 5′ domain of RNA molecule is described based on the different SAXS curves. This study suggests that CNBP binding requires multiple domains of Bvht, with a major role of RHT/AGIL RNA motif. Moreover, in a more methodological sense, it demonstrates how even low resolution data together with RNA reactivity can support the dynamic description of structure-function relationship in RNA molecules.

## 5 Computational methods for the study of RNA dynamics and flexibility

Because of the rapidly expanding interest in the RNA field, the throughput of experimental tools has rapidly become a bottleneck in our ability to quantitatively and predictively correlate RNA structure, function, and dynamics. In this respect, a variety of computational tools have been developed since the beginning of the extensive investigation of RNA spatial organization (Figure 3; Table 2). These tools provide a valuable help in interpreting the available experimental data, by adding connections between structure, dynamics, and function, and by generating experimentally testable hypotheses. If this is a longstanding process in “standard” drug discovery approaches (i.e., small molecules with few degrees of freedom binding to proteins/receptors), the complexity is higher for RNA due to its structural heterogeneity as demonstrated by spectroscopy. This fostered the development of a variety of computational techniques with different levels of precision and accuracy necessary for interpreting experimental observables from different sources. Moreover, this allowed the inclusion of population-averaged interpretation of observables, above mentioned as an asset of the most modern drug discovery methodologies, in the drug discovery practices.

MD has emerged as the most versatile technique for the integration of the available experimental techniques with numerical physical models of structure and dynamics. The main goals of atomistic MD simulations are the simultaneous simulation of structure and dynamics of RNA molecules explicitly and in real time to support the interpretation and planning of experimental measurements of such dynamics. At the same time, they provide reliable and experimentally testable predictions and insights that are not obtainable by current experimental methods. Due to the approximations required in MD simulations [see (Sponer et al., 2018) for details], experimental data can sometimes only qualitatively be reproduced or predicted, but even in these cases can be used to design/drive new experimental design.

Due to the involvement of metal centers in the RNA reactivity (e.g., enzymatic activity), MD simulations may be complemented by quantum mechanical (QM) calculations, leaving to molecular mechanical (MM) the treatment of distal layers of the RNA that are not directly involved in forming or breaking covalent bonds, to provide context and to capture the impact of conformational dynamics on the reaction (Banáš et al., 2009). Despite the high statistical quality of such studies, they cannot capture the overall dynamics of large objects in large time windows, which is the main goal of the present section of this review. Coupled classical MD and QM/MD shed light on ribozyme catalytic mechanism, which involves manganese dicationic ion (Casalino et al., 2016).

In the protein and protein-protein interactions field, standard MD simulations have been considered a gold standard methodology for the inspection of biomolecules plasticity and their role in molecular recognition, for many years. Concerning RNAs, the high heterogeneity of the transitions timescales disallows the straightforward application of the aforementioned methodologies. Thus, enhanced sampling methods are of strong interest to overcome complicated energy barriers and to efficiently explore a rough free energy surface, simulating events that occur in the range of biologically and thermodynamically relevant RNA motions timescale. Indeed, MD simulation has been recently employed to overcome the high costs and time-consuming SELEX process, as well as to study the interaction mechanism between the aptamer and the target molecule on the atomic scale (Su et al., 2021).

In general, the standard MD simulations, for the study of recognition dynamics, are relatively often used for RNA, but always coupled to other techniques like free energy perturbation or MMPBSA calculations (Chen J. et al., 2019) or steered MD (Levintov and Vashisth, 2020), the details of which are beyond the aim of the present review. Among the others, three most popular classes of enhanced sampling methods, (and here reported as of interest for the general reader) commonly used in simulative molecular disciplines are Markov state models (MSM), replica exchange simulations (RES) and metadynamics (MetaMD) (Sponer et al., 2018). As previously reported for the experimental methodologies (Section 4), we highlight the most relevant and general aspects of works in the field without specific references to the role played, either of the target or of the drug, due the low number of studies in the field that see the RNA in the role of a drug.

### 5.1 Long MD simulations and markov state models

The huge increase in the computational power available at relatively low expense allowed in the recent years the performance of extensive MD simulations that can be analyzed in terms of states and their relative population. A transition probability matrix can be constructed from MD simulations providing the probability of observing transitions between pairs of population clusters, by means of discrete jumps (Husic and Pande, 2018). The system is modeled as a Markov chain between the microstates, governed by the transition probability matrix, so that the probability distribution of the microstates at time depends only on the distribution of microstates proceeding them.

Being already successfully applied to the study of many proteins systems (Shukla et al., 2015; Sirur et al., 2016; Konovalov et al., 2021), they are now starting to gain their space also in the world of RNA-related systems. Markov State Models (MSM) have been used in a smart way to reveal that RNA polymerase II (Pol II) translocation is driven purely by thermal energy and does not require the input of any additional chemical energy. Due to the very long timescales involved in the process of translocation, the authors applied a morphing method (i.e., the Climber algorithm) to the available experimental structures of Pol II. This led to the identification of four metastable states along the Pol II translocation pathway, two already known and two newly identified metastable states, and the entire free energy landscape of the whole cyclic process alongside the populations of the states and their interconversion kinetics. This thermodynamic and kinetic characterization of the system led to the comprehension of the role of the mutual internal rearrangement dynamics of a helix and a loop (namely, BH and TL) during the entire translocation mechanism.

Few examples can be found in the literature on the application of the MSM to RNA-ligand complexes. A notable exception is constituted by the study of the structural diversity and dynamics of a theophylline-binding RNA aptamer in its unbound state (Warfield and Anderson, 2017). MSM have been used to characterize the ensemble of conformations that the aptamer adopts in the absence of theophylline and their interconversion kinetics. *Via* additional docking calculations, the theophylline binding site is found only in one ensemble of conformers accounting for one quarter of the overall population, whereas most of them are binding-incompetent, lacking a binding pocket that can accommodate theophylline. Moreover, the complete theophylline binding pathway has been simulated, supporting prior experimental observations of slow theophylline binding kinetics by showing that the binding site must undergo a large conformational rearrangement after the aptamer and theophylline forms an initial complex, namely, the rearrangement of a single base from a buried to a solvent-exposed orientation.

Another example of the application of MSM applied to RNA complex, to characterizing the RNA fraying kinetics, is reported in Pinamonti et al. (2019). Here the authors set up a protocol to study the kinetic and the thermodynamic of fraying process, and reproduce the kinetic properties of an RNA double helix in qualitative agreement with experimental data.

### 5.2 Replica exchange

In replica exchange schemes (RE) numerous replicas of the system are simultaneously simulated using different parallelization parameters (e.g., temperature or potential energy function) and, periodically, exchanges are attempted between them according to a Metropolis criterion (Metropolis et al., 1953). The most common schema employs replicas at different temperatures (T-REMD): at a higher temperature a system crosses more easily energy barriers and explores more efficiently its conformational space than a system at low temperature, consequently the ground state replica sampling is enhanced (simulated annealing principle). The replica exchange protocol can be generalized to methods where ergodicity is not obtained by increasing temperature but by scaling portions of the force field or adding penalty potentials disfavoring specific structures (e.g., by biasing/flattening potentials along selected dihedral angles). These methods are generally known as Hamiltonian replica exchange (H-REMD) methods since the different replicas use different Hamiltonian functions. They have been extensively used to characterize proteins and nucleic acids [for a more extended reference see (Sponer et al., 2018)], but only recently their application to RNA dynamics and recognition emerged in literature [e.g., in the determination of RNA stem loops dynamics in the 5′-UTR of SARS-CoV-2, (Bottaro et al., 2021)].

High temperature simulations alone have sometimes been used qualitatively to enhance sampling of RNA systems; however, they cannot be used to directly estimate the values of experimental observables at physiological temperatures. H-REMD sampling technique has been used by Pourjafar-Dehkordi and Zacharias (2021) to specifically accelerate domain motions of the Thermus Thermophilus Argonaute (TtAgo) system, a protein combined with a short microRNA (miRNAs) that can target mRNA molecules for translation inhibition or degradation and play a key role in many regulatory processes. In particular, the system has been studied in apo, guide bound, and guide/target bound states with H-REMD revealing the opening dynamics of the structure that can lead to accommodation of nucleic acids (namely DNA). The same outline has been almost followed for the characterization of the most common RNA binding domain in eukaryotes (Bochicchio et al., 2018), RRM (RNA Recognition Motif), with a comparison between free RNA (pre-miR20b) and RRM-bound RNA (Rbfox•pre-miR20b). The study has a twofold merit: methodologically it offers a quantitative comparison between NMR experimental and ensemble-averaged computed observables for the native system, providing a benchmark for the computational studies of this kind against experimental data; moreover, it proposes a very interesting and sound possibility of generating an RRM multiple mutant (R118D, E147R, N151S and E152T) able to bind RNA molecules that also contain point modifications with respect to the original template, i.e., in the present work the miR21 precursor (G28U, C30A and G33C). The last task required H-REMD simulations to eliminate any bias caused by the initial building up of the mutated structure. The study of the new complex revealed, despite the overall similarity between the two complexes, local changes in hydrogen bonding and an overall net thermodynamic stability of the system, suggesting a way to rationalize this type of molecules in a pharmaceutical perspective.

### 5.3 Metadynamics

In metadynamics (Laio and Parrinello, 2002), a bias potential is added to compensate the underlying free-energy barriers along a preselected collective variable (CV), i.e., a predefined descriptor of the molecular system capable of discriminating the transition state and of enhancing the transition probability. More recently, the idea of constructing a potential adaptively during the simulation has been added to the original formulation of metadynamics. Several methods of this type have been proposed (Sponer et al., 2018).

A recent interesting application of MetaMD has been illustrated for the characterization of the RNA targeting by Peptide Nucleic Acids (PNAs), one of the most established and efficient artificial systems for targeting DNA or RNA so far described (Verona et al., 2017). PNAs compounds are nucleic acid analogues in which the deoxyribose phosphate backbone is substituted by a polyamidic chain of N-(2-aminoethyl)-glycine units. Due to their high affinity for DNA and RNA, and to the high sequence-selectivity of their interaction with complementary nucleic acids, PNAs are widely used as probes for the recognition of specific DNA sequences and, conjugated to surfaces or to reporter groups, for diagnostic application or imaging (Nielsen, 2004). In drug development, PNAs have been used for blocking transcription of genomic DNA or for preventing translation of mRNA into proteins. PNAs:RNA duplexes stability has been evaluated, starting from the reconstruction of the free energy surfaces of a simplified single strand PNA and its chemical variant named g-PNA, demonstrating also in this case the extreme flexibility of the species and the existence of multiple conformers within 10 kcal/mol that could be accessible during the binding, some of them being already in a configuration ready to form a helix with a natural target oligonucleotide.

### 5.4 Coarse-grained modelling

Although the feasible time scales of biomolecules all-atom MD simulations have impressively increased in recent decades, and enhanced sampling methods allow deep exploration of the free energy surfaces of large molecules, there are many phenomena that still cannot be efficiently studied by atomistic MD. Biologically interesting processes, such as the folding of RNA structures or the domain motions of ribosomal subunits during translocation, can occur time scales of the order of milliseconds or longer, and might also involve millions of atoms or more. One alternative approach to all-atoms simulations is the use of simplified coarse-grained (CG) descriptions, i.e., the reduction of the degrees of freedom of the original atomistic system by grouping selected sets of atoms and representing them as a smaller set of CG particles (beads) that interact through effective energy functions. This reduction reduces the complexity of the calculation, and, in general, defines a smoother energy surface, which can be explored more quickly, allowing larger systems and longer simulation timescales. Because of the used approximations, CG models will generally only faithfully reproduce a specific set of observables in a limited region around the conditions (thermodynamic and non-thermodynamic) assumed in their parameterization. Therefore, these approaches must be used with care, and may sometimes require a redefinition of the observables of interest based on the model resolution. However, CG simulations can provide important insights that complement atomistic MD simulations or contribute to interpret experimental observations.

Many codes are currently available for performing simulations of RNA applying the coarse-grained scheme [OxDNA (Šulc et al., 2012), SimRNA (Boniecki et al., 2016), MARTINI (Souza et al., 2021)], some of them has been also recently used together with experimental data, for sampling conformational variability related to the reproduction of experimental observables [ERNWIN, Bvht (Kim et al., 2020)]. However, there is a plenty of room to apply these methods to RNA complexes. Nevertheless, parametrization of intramolecular interactions at the CG level is quite difficult, especially for protein-nucleic acid complexes, due to the low availability of experimental structures ready to be used for correctly setting up the corresponding force-field. Despite the increasing computational power at lower cost and the high quality of all the aforementioned methods, it is still impossible to simulate at atomic level large flexible systems of biological interest (e.g., lncRNAs) with high level of accuracy, which requires further development.

### 5.5 Molecular dynamics-based methods and experimental measurements

The computational dynamics of nucleic acids has been recently successfully coupled to experimental techniques to compensate their intrinsic uncertainties or to properly reconstruct structural ensembles linked to physical observables that depend on the coexistence of several states at the thermal equilibrium. The techniques that have been most efficiently coupled to MD simulations are NMR and SAXS, with a synergy that has been largely inspired by previous similar studies on protein systems. A recent example, is an application of SAXS-derived penalty functions to replica exchange MD simulations based on the maximum entropy principle (He et al., 2022). The application of this principle ensures that SAXS-driven simulations can be successfully used also in cases of extreme flexibility (in absence of a statistically dominant conformation). Interestingly, the authors propose a downstream comparison with experimental FRET data of one of the considered systems (HJH). Moreover, an extensive analysis is reported about the sensitivity of the method to detect the Mg^2+^ ions binding sites on the surface and in the RNA cavities. This topic has been recently covered by a similar study in the field (Bernetti et al., 2021) that, using MetaMD and reconstructing SAXS spectra *a posteriori*, demonstrated how explicit-solvent SAXS spectra are necessary to correctly reconstruct the ion-dependent structural ensembles. A particular computational focus is put by Chen P. C. et al. (2019) on the usage of data from multiple sources, combining information obtained by small angle scattering experiment from different sources, i.e., X-rays and neutrons, a technique that recently emerged to be increasingly important in the determination of NMR structural and dynamic organization [a multi-technique study in this sense is reported in Chen P. C. et al. (2019)]. Even if MD simulations-based refinement of SAXS data has a relatively longstanding tradition (Chen and Hub, 2015), the authors highlight the importance of integrating SANS together with SAXS to avoid overfitting problems in building ensembles of structures that are representative of experimental data and henceforth of the inner dynamics of macromolecules, especially in the study of complexes. Noteworthy, concerning the topic of the present review, the method has been proposed with a general profile for any system belonging to the soft matter field, and it includes also the refinement of the Sxl−Unr−msl2 mRNA ternary complex (SUM), that plays an important role in female *Drosophila* flies to maintain equal expression levels of X chromosome linked genes between the sexes, previously studied by X-ray crystallography and SAXS experiments and then refined using neutron scattering. As well as, combined with MD, to study aptamer LC-18, designed for the recognition of lung adenocarcinoma cells and identify its functional truncated portion (Morozov et al., 2021).

Aside this more global structural aspect of nucleic acids dynamics and molecular recognition, some recent study demonstrated that SHAPE reactivity can be efficiently used in conjunction with MD simulations. A recent work by Hurst et al. (2018) established the possibility of correlate multiple key factors, such as the nucleotide interaction strength, SHAPE reagent accessibility, and base-pairing pattern to build an analytical semiempirical function, namely the three-Dimensional Structure-SHAPE Relationship (3DSSR) function, to characterize the conformational flexibility and SHAPE reactivity based on the conformational and energetics information. Even if only conceiving a future direct involvement of SHAPE experimental data, Chen and coworkers (Ebrahimi et al., 2019) established a multiplexed method that makes usage of secondary structure restraints incorporated in a bidimensional grid of replicas, to accurate predict RNA tertiary *de novo* fold. The central philosophy of this method is to conservatively restrain only unambiguously assigned regions of RNA secondary structure to increase the efficiency of finding the most stable tertiary structure configuration. Incorporating even a small number of long-range native contacts, dramatically reduces the conformational space to be sampled by the simulation model.

## 6 Selectivity and specificity of binding for RNAs molecules

### 6.1 NMR based screening

Prior to physicochemical characterizations of intermolecular interactions, the identification of small molecules that specifically bind to the RNA of interest is of paramount importance. For metabolite sensing RNA riboswitches, cognate ligands are often identified, validated, and annotated during their biochemical characterizations. For other RNAs of interest, disease-linked regulatory RNAs, RNA-binding small molecules are often identified from a large pool of chemical libraries *via* high-throughput screening (HTS). Despite having lower throughput relative to HTS, NMR spectroscopy is also a powerful tool for identifying and validating small molecules that interact with biomolecules and has played a significant role in protein-targeted drug discovery. Excellent reviews have been published in recent years (Thompson et al., 2019), which provide thorough discussions of various NMR experiments for identifying protein-binding small molecules as well as evaluating strengths and liabilities of individual methods. Since many of these methods are based on observing ligand NMR signals, the nature of a target, whether it is a protein or an RNA, has minor influence on experimental setups of these methods, enabling their direct applications in RNA-binding small molecules identification. A recent NMR based fragment-screening approach has been employed to identify small molecules able to target SARS-CoV-2 regulatory RNA elements, known as stem loops, to develop new, and more specific, antivirals (Sreeramulu et al., 2021).

Saturation transfer difference (STD) NMR spectroscopy is one of the most widely used NMR methods in drug discovery, such as fragment-based drug discovery (FBDD) screening for protein targets. STD experiments build upon magnetization transfer between biomolecules, such as proteins or RNAs, and small ligands (Wagstaff et al., 2013). If a ligand binds the biomolecule, its NMR signals can also be saturated due to intermolecular nuclear Overhauser effect (NOE) linked to spin relaxation phenomena. In contrast, for any ligands that do not interact with the biomolecule, their NMR signals are minimally affected by the irradiation of biomolecular NMR signals. The difference between saturated and unsaturated spectra reveals the ligands that bind the biomolecule. STD experiment can efficiently screen a pool of small molecules and identify binding-competent ligands. Despite its wide usage for proteins, STD is less viable in screening RNA-binding small molecules, but it has been successfully employed to characterize RNA-binding small molecules (Mayer and James, 2002; Vasile et al., 2014; McRae et al., 2020).

WaterLOGSY (water-ligand observed *via* gradient spectroscopy) is a ligand-observed NMR technique that can be used in target-directed drug screening or ligand validation to assess the binding of molecules to macromolecules (Calabrese et al., 2019). WaterLOGSY effects are achieved by applying indirect magnetic saturation of the macromolecule by selective saturation of bulk water protons. Magnetization is first transferred from water to labile (exchangeable) RNA protons, that are proximal to the ligand binding site and then to the compounds interacting with the RNA. WaterLOGSY has been widely used for detecting macromolecule-ligand interactions for RNA, DNA, and proteins because it is efficient and easy to interpret also in the context of ligands ranking among libraries. WaterLOGSY is very fast and requires a low RNA quantity. Moreover, WaterLOGSY experiments do not require RNA or small molecule labeling. Compared to small RNAs, larger RNA structures have longer rotational correlation times which results in a more efficient magnetization transfer, leading to stronger signals. (Thompson et al., 2019).

### 6.2 SHAPE

In fragment-based ligand discovery one or more small-molecule “fragments” of low molecular mass (200–400 Da) and low to moderate affinity are identified that bind a target of interest, and these fragments are then either elaborated or linked to create more effective ligands (Erlanson et al., 2004). Fragment-based ligand discovery can be also performed *in silico*, further reducing time and costs of ligand building (Warner et al., 2014; Li, 2020). This method has been successfully employed to identify initial hit compounds for RNA binding, however to date, fragment-based methods are not used to create a high-affinity RNA-targeting compound *de novo*.

Zeller and coworkers have recently shown that many RNAs bind their ligands *via* multiple subsites, which are regions of a binding pocket that contact a ligand in an independent or cooperative manner (Zeller et al., 2022b). Besides high-affinity RNA binding can occur even when subsite binding shows only modest cooperative effects and when the linking coefficient is unfavorable. Identification of multiple fragments that bind the same RNA would make it possible to take advantage of potential additive and cooperative interactions between fragments within the binding pocket.

Zeller et al. (2022a) developed a technology that leverages fragment-based screening and SHAPE-MaP RNA structure probing to discover small-molecule fragments that bind an RNA structure at roughly nucleotide resolution. A modular RNA screening, tested on the thiamine pyrophosphate (TPP) riboswitch, has been developed implementing SHAPE as a high-throughput assay for readout of ligand binding. The construct was designed to contain two target motifs to ensure internal mutual activity control. i.e., a pseudoknot from the 5′-UTR of the whole genome (dengue) and the TPP riboswitch aptamer domain. These two structures were connected by a six-nucleotide linker, designed to be single stranded, to allow the two RNA structures to remain structurally independent. Fragments that bound to both RNA structures were easily identified as nonspecific binders. Fragment hits are identified as multiple, statistically significant differences in SHAPE reactivities. 41 fragments out of 1,500 tested has been identified as binders and further characterized by isothermal titration calorimetry (ITC) to determine binding affinities for an RNA corresponding just to the target motif. Structure-activity relationship and ITC information eventually led to a linked-fragment ligand with no resemblance to the native binders and with high ligand efficiency and drug-likeness, that binds to the TPP riboswitch with high nanomolar affinity and that modulates RNA conformation during co-transcriptional folding.

### 6.3 SELEX

Aptamers are generated using an iterative selection process that partitions oligonucleotides based on their binding or functional/catalytic activities through a process of directed chemical evolution called SELEX (Urak et al., 2016). A typical SELEX experiment consists of the enrichment of highly specific nucleotide sequences. The first step is to synthesize a random sequence DNA library (10^12^–10^15^) of ∼20–100 nucleotides containing flanking constant sequences required for PCR amplification. For RNA SELEX (Urak et al., 2016), the single stranded RNA library for each round of selection is prepared by *in vitro* transcription of dsDNA templates using T7 RNA polymerases. Modified nucleotide triphosphates (NTPs) are typically incorporated during the selection process to reduce the possibility of post-selection modifications impairing aptamer function (Urak et al., 2016). Recent breakthroughs in SELEX are the application of NGS technologies which, together with bioinformatics analysis, expedite the identification of finally selected sequences and allow tracking of aptamer evolution also in cells (cell-SELEX) (Sefah et al., 2010).

Small molecules are attractive targets for aptamer selection, as these aptamers can be used as biosensors, as recognition modules in riboswitches or even as antidotes in drug usage. However, unlike for larger complexes such as proteins, the selection of small molecule aptamers has always been challenging due to the limited number of interaction moieties for chemical immobilization of baits on a matrix and the highly denaturing conditions (e.g., extreme pH, use of solvent etc.) of chemical immobilization that may compromise the target molecule before even starting the selection process. The difficulties in the selection of small molecule binding aptamers, can be avoided by a target-immobilization free protocol called Capture-SELEX (Boussebayle et al., 2019), where the roles of pool and target are inverted. The aptamer pools are immobilized through a capture-oligonucleotide used as an anchor. To elute aptamers from their support, the free ligand molecule is incubated with the immobilized pool, thus undocking aptamers from the capture-oligonucleotide. As a result, only aptamers able to bind the original unmodified free ligand are generated.

### 6.4 SAXS

SAXS-based RNA screening (SAXScreen) procedure has been used to categorizes ligand titrations by computing pairwise agreement between scattering curves and by estimating affinities through the quantification of complex formation as deviation from the linear combination properties of solution SAXS (Chen et al., 2018). The resulting workflow for SAXScreen ranks putative interactions based only on intensities. To reduce RNA synthesis costs, it has been assumed that all ligands share a comparable binding mechanism, hence all the screening process has been based on the minimization of a single cost function containing weighted signal intensities of all the chemical species involved in the complex formation. This translates into an immediate and easy identification of the processes that deviate from a two-state binding and into a simultaneous curve fit, which produces a final ligand ranking based on dissociation constant, aside the recording of particle volume, i.e., radius of gyration. The optimized protocol allows up to 1,000 measurements per day, corresponding to 100 titrations depending on the desired precision of affinity estimation. An increase of 1–2 orders of magnitude is expected in the near future with improvements to beamline setups aimed at reducing downtime between measurements.

### 6.5 Docking

To overcome the difficulties to predict a reliable three-dimensional conformation of nucleic acids and to rapidly analyze their binding to target molecules, computational docking is a rapid and low-cost method to screen potentially interesting molecules. Nevertheless, docking of RNA molecules is different to docking of small molecules to proteins. First, the flexibility is an important component of the binding process and usually in docking simulations protein and ligand are considered as rigid, to speed up the computation. MORDOR is (probably the only) one example of induced-fit binding *via* flexible-RNA and flexible-ligand docking (Guilbert and James, 2008). Besides, RNA is a negatively charged molecule and charged ions are components of the system; ions need correctly parametrized force fields to be properly simulated. Force fields specific for nucleic acids are used in MD but they are not easily included in docking software. The electrostatic potential distinguishes proteins from nucleic acids, whereas docking software implement electrostatic potentials peculiar for protein-ligand complexes. Scoring functions specific for RNA-ligand have been developed since 2004 (RiboDock, DrugScoreRNA, rDOCK, etc.) (Morley and Afshar, 2004; Pfeffer and Gohlke, 2007; Ruiz-Carmona et al., 2014), until recently NLDock, RLDock, AnapuRNA and LigandRNA (Philips et al., 2013; Sun et al., 2020; Feng Y. et al., 2021; Stefaniak and Bujnicki, 2021), all of them are empirical or knowledge-based scoring functions. For this reason, the knowledge of structural information based on crystallographic data alone and in complex is fundamental, also to understand the features of this type of interaction.

Actually, just some examples of RNA-protein docking are available; Chauvot de Beauchene group used a fragment-based approach and docking to design *ab initio* a ssRNA (Chauvot de Beauchene et al., 2016), and Guihot-Gaudeffroy group applied known tools to inspect RNA-protein complexes (Guilhot-Gaudeffroy et al., 2014); *ad hoc* scoring function are under development (Pérez-Cano et al., 2017). Besides only a few examples of RNA -RNA docking (Yan et al., 2018), could be useful, at the present stage, to improve the development of therapeutic RNAs.
